# Generation and Characterization of a JAK2V617F-Containing Erythroleukemia Cell Line

**DOI:** 10.1371/journal.pone.0099017

**Published:** 2014-07-18

**Authors:** Wanke Zhao, Kang Zou, Taleah Farasyn, Wanting Tina Ho, Zhizhuang Joe Zhao

**Affiliations:** 1 Department of Pathology, University of Oklahoma Health Sciences Center, Oklahoma City, Oklahoma, United States of America; 2 Oklahoma School of Science and Mathematics, Oklahoma City, Oklahoma, United States of America; Southern Illinois University School of Medicine, United States of America

## Abstract

The JAK2V617F mutation is found in the majority of patients with myeloproliferative neoplasms (MPNs). Transgenic expression of the mutant gene causes MPN-like phenotypes in mice. We have produced JAK2V617F mice with p53 null background. Some of these mice developed acute erythroleukemia. From one of these mice, we derived a cell line designated J53Z1. J53Z1 cells were stained positive for surface markers CD71 and CD117 but negative for Sca-1, TER-119, CD11b, Gr-1, F4/80, CD11c, CD317, CD4, CD8a, CD3e, B220, CD19, CD41, CD42d, NK-1.1, and FceR1. Real time PCR analyses demonstrated expressions of erythropoietin receptor EpoR, GATA1, and GATA2 in these cells. J53Z1 cells grew rapidly in suspension culture containing fetal bovine serum with a doubling time of ∼18 hours. When transplanted into C57Bl/6 mice, J53Z1 cells induced acute erythroleukemia with massive infiltration of tumor cells in the spleen and liver. J53Z1 cells were responsive to stimulation with erythropoietin and stem cell factor and were selectively inhibited by JAK2 inhibitors which induced apoptosis of the cells. Together, J53Z1 cells belong to the erythroid lineage, and they may be useful for studying the role of JAK2V617F in proliferation and differentiation of erythroid cells and for identifying potential therapeutic drugs targeting JAK2.

## Introduction

Ph- myeloproliferative neoplasms (MPNs) are clonal hematopoietic malignancies in which one or more myeloid lineages are abnormally amplified. These diseases represent a group of chronic conditions including polycythemia vera (PV), essential thrombocythemia (ET), and primary myelofibrosis (PMF) [1], [2]. MPNs mainly affect older people and have an average onset age of 55 years. Complications associated with MPNs include the development of acute leukemia as well as thrombosis, hemorrhage, and myeloid metaplasia. JAK2V617F, a mutant form of tyrosine kinase JAK2, represents a major molecular defect in these diseases and is found in over 95% of PV and over 50% of ET and PMF cases [3]–[8]. Studies demonstrated that JAK2V617F has enhanced tyrosine kinase activity, causes constitutive activation of down-stream signal transducers when expressed in cells [7], and produces MPN-like phenotypes in transgenic and knock-in mice [9]–[15]. In earlier studies, we generated JAK2V617F transgenic mice by using the *vav* gene promoter which drives the transgene expression in the hematopoietic system. The transgenic mice display MPN-like phenotypes with much increased numbers of red blood cell and platelets [9]. The constitutive activation nature of JAK2V617F makes it a potential oncoprotein. In searching for other gene mutations that collaborate with JAK2V617F to drive leukemia cell transformation, we recently found that JAK2V617F and loss-function mutation of tumor suppressor p53 co-exist in two well-studied leukemia cell lines, namely, HEL and SET2 [16]. This suggests that JAK2V617F is able to drive leukemic transformation when the function of tumor suppressor p53 is lost. We then crossed JAK2V617F transgenic mice with p53 knockout mice and generated JAK2V617F mice with p53 null background. Interestingly, these mice developed acute leukemia. From one of these mice we derived an erythroleukemia cell line which we designated J53Z1. This study reports some basic feature of this cell line.

## Materials and Methods

### Materials

Antibodies for flow cytometric analysis of cell surface markers were from BD Biosciences and eBioscience. Antibodies against signaling proteins, including phospho-ERK1/2, phospho-Akt, and phospho-STAT5, were from Cell Signaling Technology. JAK2 inhibitors AZD1480 and ruxolitinib were purchased from Chemietek. All other protein kinase inhibitors were from the Approved Oncology Drugs Set IV of NCI Chemotherapeutic Agents Repository.

### Mice

Line A JAK2V617F transgenic mice which carry 13 copies of the JAK2V617F transgene were used in this study as previously described [9]. These mice have been crossed with wild type C57BL/6 mice for over 10 generations [17]. Wild type C57BL/6 and p53 knockout mice (strain name B6.129S2-*Trp53^tm1Tyj^*/J) were purchased from The Jackson Laboratory [18]. JAK2V617F mice with p53 null background were generated by crossing JAK2V617F transgenic mice with p53 knockout mice. Implantation of cultured cells into wild type C57BL/6 mice was carried out through retro-orbital injections under isoflurane anesthesia. Animals were housed in ventilated cages under standard conditions. This study was carried out in strict accordance with the recommendations in the Guide for the Care and Use of Laboratory Animals of the National Institutes of Health. The protocol was approved by the Institutional Animal Care and Use Committee of the University of Oklahoma Health Sciences Center.

### Cell culture

Bone marrow cells (1×10^−6^) were collected from a JAK2V617F/p53^-/-^ mouse and cultured in Iscove's modified Dulbecco's medium (IMDM) supplemented with 20% fetal bovine serum, and 20 µM 2-mercaptoethanol at 37°C with 5% CO_2_. The culture was maintained for three weeks with equal volumes of fresh medium added every 3–4 days. Cells were then subjected to colony culture in semisolid medium containing 1% methylcellulose. Colonies were picked after 7 days of culturing and further expanded in the liquid medium. A total of 12 clones were analyzed, and each showed essentially similar morphology and cell surface markers (see below) typical for erythroleukemia cells. Clone no. 1 was selected for detailed characterization as described below. HCD-57 erythroleukemia cells were obtained from Dr. Maurice Bondurant, Vanderbilt University, and cultured in the above liquid medium plus 1 unit/ml of erythropoietin (EPO) [19]. MV-4-11 leukemia cells (ATCC CRL-9591) were obtained from ATTC and maintained in IMDM containing 10% fetal bovine serum. Cell concentrations were determined by flow cytometric analyses. Dead cells were excluded by staining with 7-amino-actinomycin D, and CountBright absolute counting beads (Invitrogen) were used as a standard for cell counting.

### DNA extraction and PCR

Genomic DNAs were purified from mouse tails and cells by using the phenol/chloroform method after proteinase K digestion and PCR-amplified as previously described [9], [16], [20]. Genotyping of p53 knockout mice and cells was performed with a primer set containing 5′-ACAGCGTGGTGGTACCTTAT, 5′-TATACTCAGAGCCGGCCT, and 5′-CTATCAGGACATAGCGTTGG. The expected PCR products were 650bp for the targeted allele and 450 bp for the wild type allele. The JAK2V617F transgene was detected by using 5′-TACAACCTCAGTGGGACAAAGAAGAAC and 5′-CCATGCCAACTGTTTAGCAACTTCA with an expected PCR product of 594bp. Endogenous mouse Jak2 was detected by using 5′-AGACTTCCAGAACCAGAACAAAG and 5′-TCACAGTTTCTTCTGCCTAGCTA which gave rise to an 84bp PCR product. PCR products were analyzed on 1.5% agarose gels and visualized by ethidium bromide staining.

### Total RNA isolation and real time PCR analysis

Total RNAs were isolated from cultured cells and mouse tissues by using the RNeasy Mini kit (Qiagen), and single strand cDNAs were synthesized with equal amounts of total RNAs by using the QuantiTect reverse transcription kit from Qiagen. Real time PCR was performed with iQ SYBR Green Supermix (Bio-Rad) and primers specific for transgenic human JAK2V617F, mouse Jak2, glyceraldehyde-3-phosphate dehydrogenase (GAPDH), GATA1, GATA2, and erythropoietin receptor EpoR. Melting curves were analyzed to confirm specific amplification of desired PCR, and the identities of final PCR products were verified by separation on agarose gels. For quantification, standard curves were obtained by performing PCR with serial dilutions (covering 5 orders of magnitudes) of purified PCR products in salmon sperm DNA [21]. Levels of transcripts were normalized against that of GAPDH.

### Cell and tissue staining

For Wright-Giemsa staining, cells were spun onto glass slides by cytocentrifugation. For histological analysis, tissues were fixed in formaldehyde and embedded in paraffin. Tissue sections (5 µm) were deparaffinized and then stained with Hematoxylin and eosin (H&E). Images were captured by using a DP71 digital camera attached to an Olympus BX51 microscope.

### Flow cytometric analyses

Cells were stained with fluorescein isothiocyanate (FITC)-, phycoerythrin (PE)-, or allophycocyanin (APC)-conjugated monoclonal antibodies specific for mouse CD71 (clone C2), CD117 (clone 2B8), Sca-1 (clone D7), TER-119 (clone Ter-119), CD11b (clone M1/70), Gr-1 (clone RB6-8C5), F4/80 (clone BM8), CD11c (clone N418), CD317 (clone eBio927), CD4 (clone RM4-5), CD8a (clone 53-6.7), CD3e (clone 145-2C11), B220 (clone RA3-6B2), CD19 (clone 1D3), CD41 (clone MWReg30), CD42d (clone 1C2), NK-1.1 (clone PK136), and FceR1 (clone MAR-1) (from BD Biosciences and eBioscience). Stained cells were washed and analyzed by 4-color flow cytometry on a FACSCalibur flow cytometer (BD Biosciences) at the Flow and Image Cytometry Laboratory of University of Oklahoma Health Sciences Center. Data were collected by using the Cell Quest software (BD Biosciences) and analyzed by using the Summit software (Dako Colorado, Inc.). At least 15,000 total events were analyzed. Dead cells were excluded according to staining with 7-amino-actinomycin D. For apoptosis analysis, the cells were stained with FITC-Annexin V and propidium iodide.

### Cell stimulation and western blot analyses

J53Z1 cells were incubated in plain IMDM for 4 hours and then stimulated with EPO (10 U/ml) or mouse stem cell factor SCF (50 ng/ml) for 10 min. Cells were collected in and washed with ice-cold phosphate-buffered saline (PBS). Protein samples were prepared by adding SDS gel sample buffer directly into the cell pellets. Western blotting analyses were performed with antibodies against phospho-ERK1/2, phospho-Akt, and phospho-STAT5 followed by horseradish peroxidase-conjugated secondary antibodies. Captures of enhanced chemiluminescence signals were done by using FluorChem SP imaging system from Alpha Innotech.

### Inhibitor screening and cell survival assays

J53Z1 cells were cultured in the presence of various tyrosine kinase inhibitors. After 72 hours of culture, cell viability was assessed by performing XTT assays with XTT and phenazine methosulfate following standard protocols [22].

### Statistical analysis

Statistical analyses were performed using the Excel program. Differences between 2 groups of samples were assessed using *t* tests. P values less than 0.05 (2-tailed) are considered significant.

## Results

### Generation of the J53Z1 cell line

**Figure 1:**
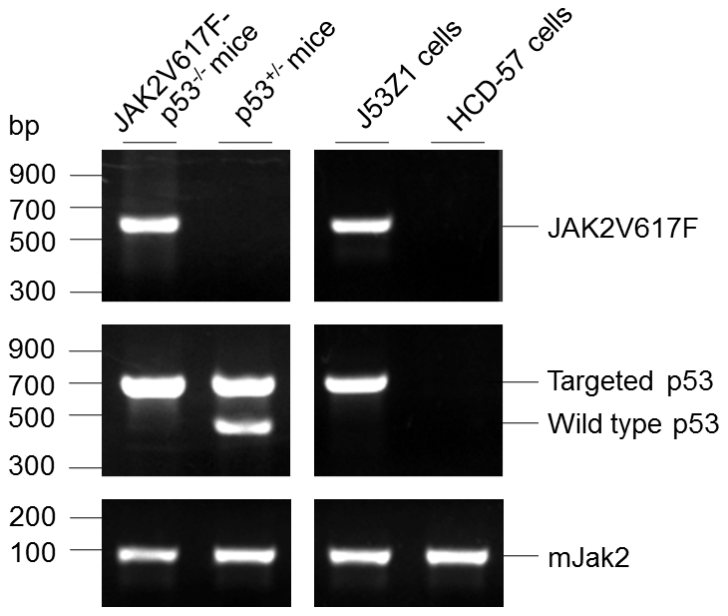
Genotyping of JAK2V617F/p53^-/-^ mice and J53Z1 cells. Genomic DNAs isolated from mouse tails and cultured cells. JAK2V617F transgene, wild type 53, mutant p53, and endogenous mouse Jak2 were PCR-amplified with specific primers described in Materials and Methods. The PCR products were resolved on 3% agarose gels and visualized by ethidium bromide staining. HCD-57, a previously established mouse erythroleukmia cell line, was analyzed for comparison.

By crossing JAK2V617F transgenic and p53 knockout mice, we generated JAK2V617F/53^-/-^ mice (Figure 1). It has been reported that JAK2V617F mice developed MPN-like phenotype in two months while homozygous p53^-/-^ develop tumors (principally lymphomas and sarcomas) at three to six months of age [9], [18]. Interestingly, we found that JAK2V617F/53^-/-^ mice died of apparent acute leukemia in 4 months with huge spleen and liver. Detailed characterization of these mice will be reported elsewhere. Bone marrow cells from one of these mice were collected and cultured in IMDM medium supplemented with 20% fetal bovine serum. Cell proliferation took off in 3 weeks as a single cell suspension. We then performed colony culture in semisolid medium containing 1% methylcellulose. A total of 12 clones were analyzed, and each showed essentially similar morphology and cell surface markers typical for erythroleukemia cells (see below). Clone no. 1 was selected for detailed characterization as described below. We designated the new cell line J53Z1. JAK2V617F and p53 genotyping verified the presence of JAK2V617F and the absence of wild type p53 in the cells as seen in the parental JAK2V617F/p53^-/-^ mouse (Figure 1). For comparison, we also analyzed a JAK2V617F-negative p53^+/-^ mouse and a previously established erythroleukemia cell line, namely, HCD-57 [23]. As expected, HCD-57 cells do not contain JAK2V617F. Interestingly, however, they did not give rise to any p53 PCR product either. The primers used for amplification of p53 cover intron 6 and exon 7 of the mouse p53 gene. This likely indicates the p53 gene is lost in HCD-57 cells. Indeed, reverse-transcription PCR failed to amplify any p53 transcripts, and no p53 protein was detected by using anti-p53 antibodies (not shown). Finally, as a control, mouse Jak2 was amplified to verify the quality of DNA used for genotyping of JAK2V617F and p53 (Figure 1, bottom panel).

### Proliferation and morphology of J53Z1 cells

**Figure 2:**
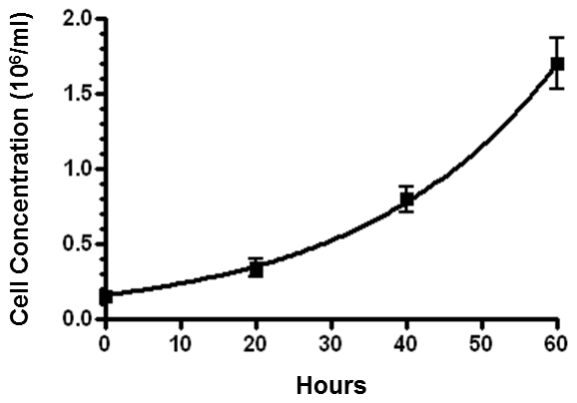
Growth curves of J53Z1 cells in suspension culture. J53Z1 cells were grown in IMDM medium supplemented with 20% fetal bovine serum. Cell numbers were counted by using flow cytometery as described in Materials and Methods. Error bars denote standard deviation (n = 3).

**Figure 3:**
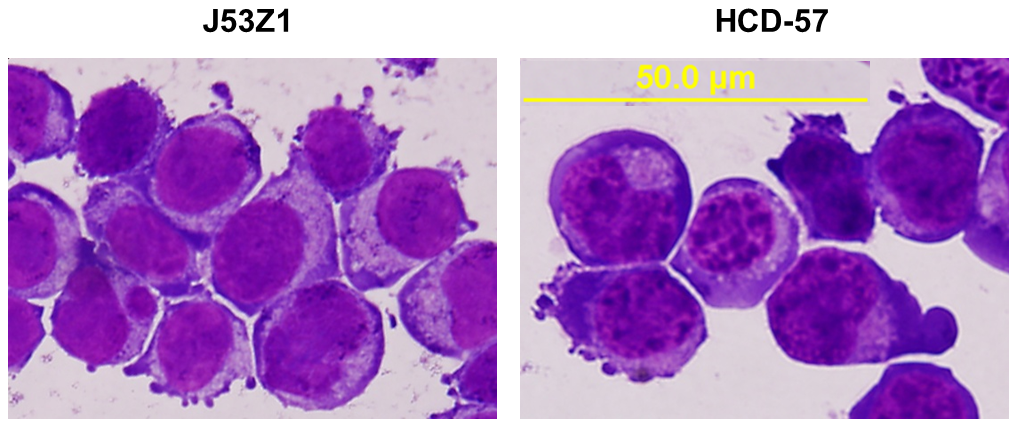
Morphology of J53Z1 cells. Normal growing J53Z1 cells were attached to glass slides by cytospin and then subjected to Wright-Giemsa staining. HCD-57 erythroid leukemia cells were stained for comparison. Photos were taken with a 100x objective lens.

J53Z1 cells proliferated rapidly in suspension culture with a doubling time of ∼18 hr (Figure 2). At present, the cells have continued to grow in liquid medium for 14 months under the same culture condition, showing no changes in morphology and proliferation rate. Theoretically, they have grown over 560 generations. We have made frozen stocks of cells with 10% dimethyl sulfoxide (DMSO) and cryopreserved them in liquid nitrogen. After 12 months of cryopreservation, we were able to recover the cells readily. The growth of J53Z1 cells were significantly retarded in serum-free media, and the cells eventually died in two weeks. Treatment with erythropoietin (EPO), stem cell factor (SCF), DMSO, and phorbol-12-myristate-13-acetate (PMA) failed to induce differentiation of the cells. We have thus generated a new JAK2V617F-positive and p53^-/-^ cell line. Figure 3 illustrates the morphology of J53Z1 cells together with HCD-57 erythroleukemia cells after Wright-Giemsa staining. Like HCD-57 cells, J53Z1 cells were mostly round to ovoid with a modal diameter of 13 to 20 µm. They displayed large nuclei with a few prominent nucleoli and basophilic cytoplasms. Cytoplasmic vacuoles and protrusions are also present in some cells. These cells showed morphologic characteristics of erythroblasts.

### Expressions of erythroid proteins in J53Z1 cells

**Figure 4:**
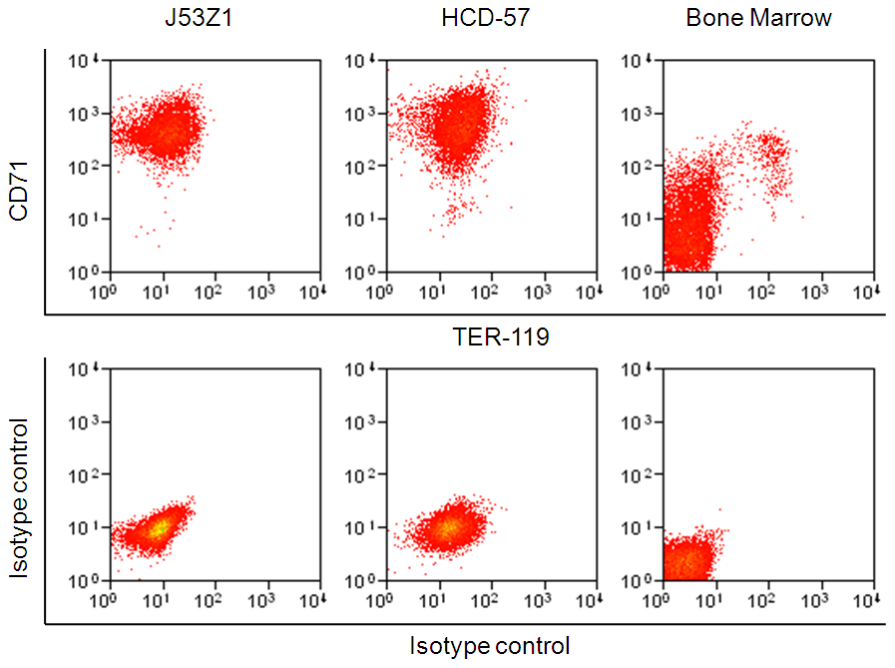
Flow cytometric analysis of CD71 and TER-119 expression on J53Z1 cells. J53Z1, HCD-57, and normal mouse bone marrow cells were labeled with anti-CD71 and TER-119 monoclonal antibodies or with a nonspecific isotype control mouse IgG before flow cytometric analysis.

**Figure 5:**
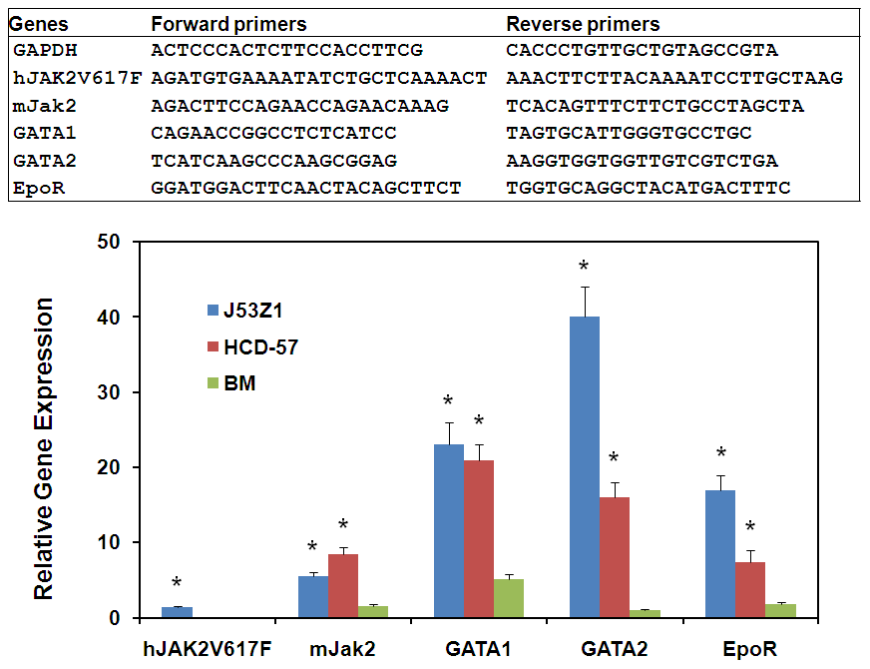
Real time PCR assays of gene expression in J53Z1 cells. Expressions of indicated genes were analyzed by real time PCR using specific PCR primers shown in the top panel. Data represent relative mRNA levels (mean±SD, n = 3) normalized to mouse GAPDH which was defined as 1000. HCD-57 and normal mouse bone cells were analyzed for comparison. *P<0.001 in reference to normal bone marrow cells.

Flow cytometric analyses of cell surface markers further verified the erythroid lineage of J53Z1 cells. Like HCD-57 cells, J53Z1 cells are strongly positive for immature erythroid cell marker CD71 but negative for TER-119, and the level of CD71 is comparable to that seen in HCD-57 cells and higher than the level observed in normal mouse bone marrow erythroid cells (Figure 4). J53Z1 cells are also positive for expressions of CD117 (cKit) but are negative for hematopoietic stem cell marker (Sca-1), myeloid cell markers (CD11b and Gr-1), monocyte marker (F4/80), dendritic cell markers (CD11c and CD317), T-cell markers (CD4, CD8a, and CD3e), B-cell marker (B220 and CD19), megakaryocyte markers (CD41and CD42d), NK cell marker (NK-1.1), and mast cell marker (FceR1) (see Table 1). Erythroblasts are divided into various stages based on CD71 and TER-119 expression [24]. Since J53Z1 cells were stained negative for TER-119, we believe that they are arrested at a very earlier stage before proerythroblasts during erythroid development. We further employed real time PCR to determine the expressions of GATA1, GATA2, EpoR, mouse Jak2, and transgenic human JAK2V617F in J53Z1 cells (Figure 5). Normal mouse bone marrow and HCD-57 cells were analyzed for comparison. As expected, expressions of EpoR and mouse Jak2 were found in both J53Z1 and HCD-57 cells, at levels above those seen in normal bone marrow cells. In addition, the expression of transgenic human JAK2V617F was detected in J53Z1 cells only, at a level below that of mouse Jak2, which is consistent with its relative expression level in hematopoietic tissues from JAK2V617F transgenic mice [9], [17]. Interestingly, substantial expressions of both GATA1 and GATA2 were observed in J53Z1 and HCD-57 cells, at levels much higher than those seen in total mouse bone marrow cells. The GATA family transcription factors have distinct and essential roles in hematopoiesis [25]. As a master regulator of hematopoietic differentiation, GATA1 is expressed in erythroid cells and megakaryocytes. In contrast, GATA2 is essential for maintenance of the hematopoietic stem cell compartment and is also involved in the initial activation of GATA1 expression at the first steps of erythroid/megakaryocytic differentiation. During erythroid differentiation, expressions of GATA1 and GATA2 are reciprocal thereby forming the so-called GATA switch [25]. Expression of both GATA1 and GATA2 in J53Z1 and HCD-57 cells suggest that these cells are arrested at a stage that GATA1 and GATA2 expressions overlap. These cells thus represent a unique stage of erythroid development.

**Table 1:** Surface Markers on J53Z1 Cells.

Markers	Positivity	Markers	Positivity
CD71	high	CD4	negative
CD117 (cKit)	high	CD8a	negative
Sca-1	negative	CD3e	negative
TER-119	negative	B220	negative
Gr-1	negative	CD19	negative
CD11b	negative	CD41	negative
F4/80	negative	CD42d	negative
CD11c	negative	NK1.1	negative
CD317	negative	FceR1	negative

### Response of J53Z1 cells to growth factors

**Figure 6:**
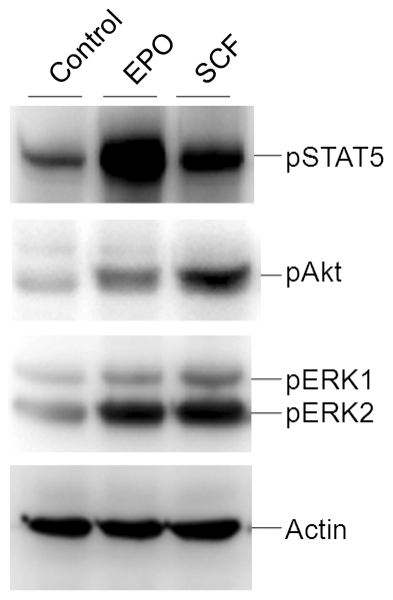
Response of J53Z1 cells to growth factors. J53Z1 cells were serum-starved for 5 hr and then stimulated with 10 Units/ml EPO or 50ng/ml SCF for 10 min. Cell extracts were analyzed for activation of indicated signaling proteins by using phospho-specific antibodies. Equal protein loading was demonstrated by blotting with anti-actin.

Primary erythroid progenitor cells rely on two key growth factors, namely, EPO and SCF, for survival and expansion [26]. Although J53Z1 cells are immortal and become independent of specific growth factors, they may still be responsive to these factors. Indeed, when serum-starved J53Z1 cells were treated with EPO and SCF, strong phosphorylation of ERK1/2, Akt, and STAT5 were observed (Figure 6). The EPO- and SCF-induced signal transduction is consistent with the expression of the EPO receptor EpoR and the SCF receptor CD117 (cKit) revealed by real time PCR and flow cytometry, respectively (Figure 5 and Table 1). The data provided further evidence that J53Z1 cells belong to the erythroid lineage. J53Z1 cells should serve as a good cell system for studying cell signaling involved in erythroid development.

### Induction of acute leukemia in mice implanted with J53Z1 cells

**Figure 7:**
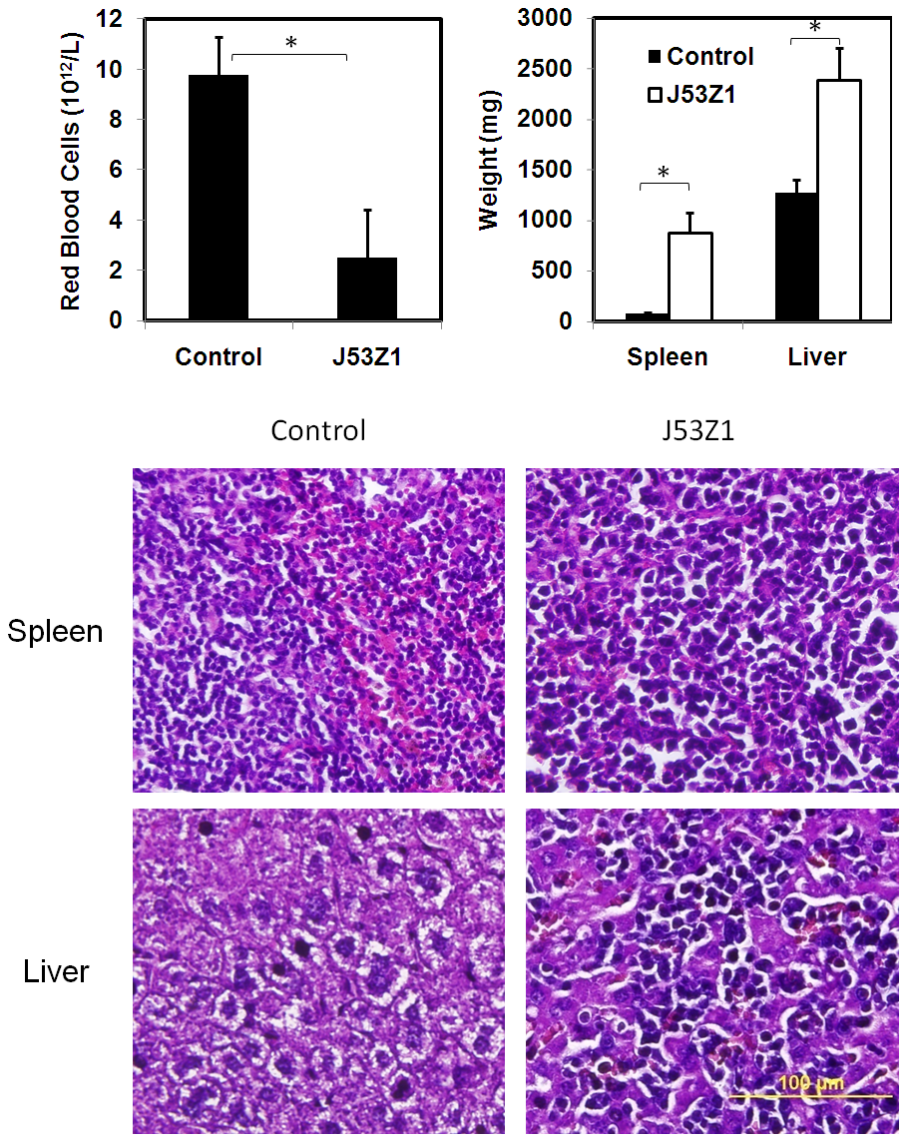
Development of erythroleukemia phenotypes in mice receiving implantation of J53Z1 cells. Cultured J53Z1 cells (1×10^6^) were implanted into 12-week-old wild type C57Bl/6 mice through retro-orbital injections. **Upper panel.** Red blood cells, spleen, and liver were analyzed 2 to 4 weeks after implantation. Error bars denote standard deviation (n≥4), *P<0.001. **Lower panel.** Paraffin sections of spleen and liver from representative control and J53Z1-implanted mice were subjected to H&E staining. Note the loss of normal tissue architecture and infiltration of densely stained erythroleukemia cells in tissues from J53Z1-transplanted mice. Photos were taken with a 40x objective lens.

J53Z1 cells were derived from a mouse with acute erythroleukemia. To find out if they still possess the ability to cause leukemia, we implanted 1×10^6^ cultured J53Z1 cells into wild type C57Bl/6 mice through retro-orbital injections. The recipient mice developed anemia and died within 4 weeks with enlarged spleen and liver (Figure 7, upper panel). H&E staining revealed massive accumulation of erythroblast cells in the liver and spleen, characteristic of erythroleukemia (Figure 7, lower panel). This indicates that J53Z1 cells contain cancer stem cells that can initiate cancer *in vivo*.

### Inhibition of J53Z1 cells by selective tyrosine kinase inhibitors

**Figure 8:**
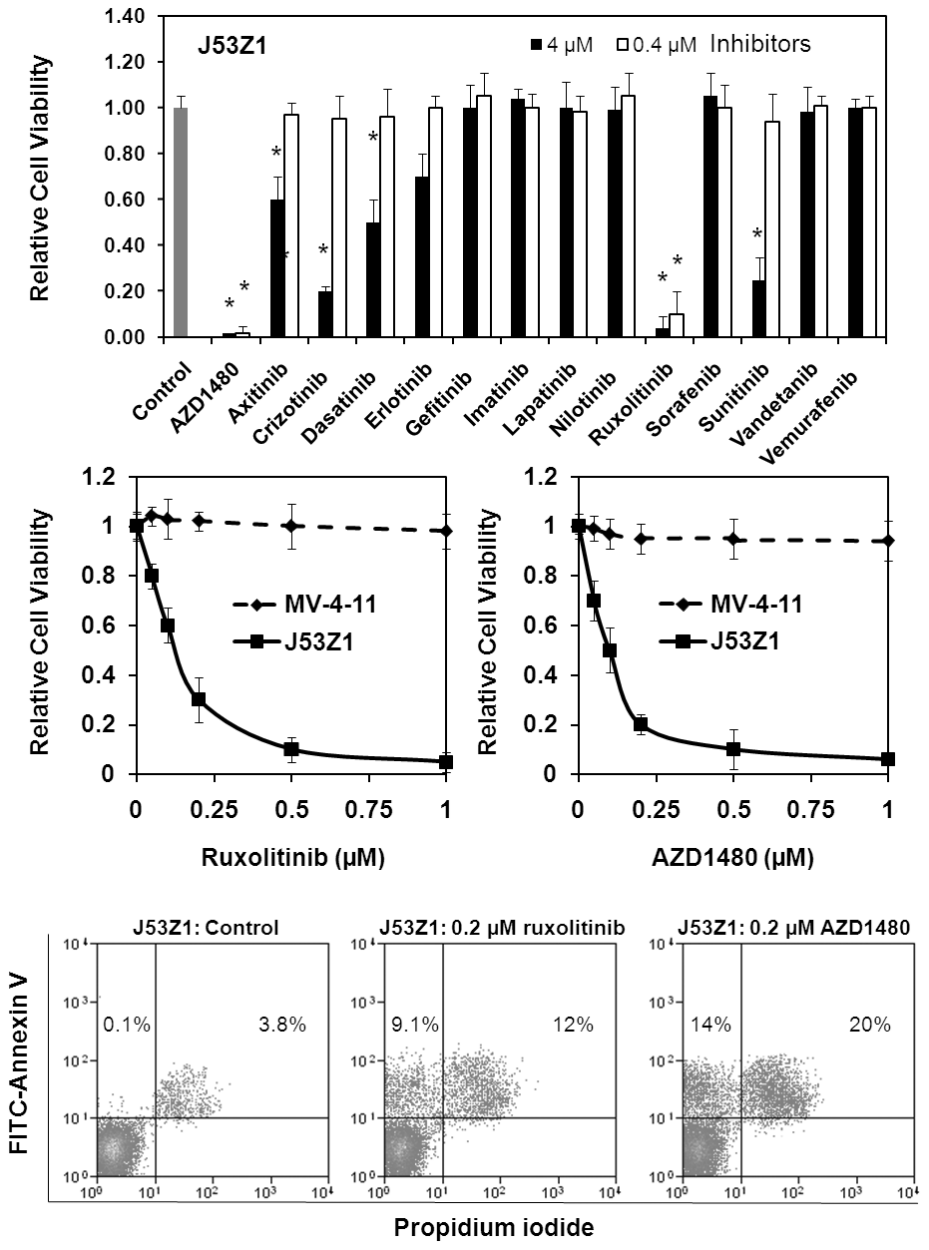
Inhibition of J53Z1 cells by selective protein kinase inhibitors. J53Z1 cells were cultured in the presence of various concentrations of indicated protein kinase inhibitors. **Top and middle panels.** Cell viability was assessed by XTT assays after 72 hr of incubation. Control experiments were performed in the presence of 0.1% DMSO. Error bars denote standard deviation (n = 3). *P<0.001 in reference to control. Note that MV-4-11 cells were analyzed for comparison (middle panel). **Bottom panel.** Apoptosis assays were performed with J53Z1 cells after 24 hr of incubation with 0.2 µM of ruxolitinib or AZD1480. Cells were stained with FITC-annexin V and propidium iodide. Percentages of annexin V-positive cells are indicated.

As a constitutively active tyrosine kinase, JAK2V617F likely plays a crucial role in supporting the proliferation of J53Z1 cells. Naturally, inhibition of its kinase activity should block cell growth. We analyzed a total of 14 protein kinase inhibitors for their inhibitory effects on J53Z1 cells as shown Figure 8. Except for AZD1480 which is a JAK2 inhibitor currently in clinical trials [27], the rest are FDA-approved anti-cancer drugs. Interestingly, at 4 µM, several inhibitors including AZD1480, axitinib, crizotinib, dasatinib, erlotinib, ruxolitinib, and sunitinib showed inhibitory effects on the growth of J53Z1 cells, while gefitinib, imatinib, lapatinib, nilotinib, sorafenib, vandetanib, and vemurafenib showed no effects. However, when the concentration was reduced to 0.4 µM, only AZD1480 and ruxolitinib remained strongly inhibitory. More detailed analyses revealed that AZD1480 and ruxolitinib inhibited J53Z1 cells with IC50 values of 0.10 and 0.14 µM, respectively. In contrast, they displayed no inhibitory effects on MV-4-11 leukemia cells which carry a FLT3-ITD mutation [28], [29]. The results are quite expected since AZD1480 is known to be a potent, selective JAK2 inhibitor [27], while ruxolitinib is a potent JAK1 and JAK2 inhibitor approved by FDA for treatment of myelofibrosis [30]. It should be noted the inhibitory effects of other kinase inhibitors on J53Z1 cells at the higher concentration may also be attributed to the inhibition of JAK2V617F. In fact, our early studies have demonstrated that EGFR inhibitor erlotinib but not gefitinib inhibits JAK2 [31]. On the other hand, sunitinib is considered a broad range tyrosine kinase inhibitor, and it should not be surprising if it inhibits JAK2 also. Indeed, our in vitro kinase assays by using isolated JAK2 kinase demonstrated that axitinib, crizotinib, dasatinib, and sunitinib but not imatinib, lapatinib, nilotinib, sorafenib, vandetanib, or vemurafenib had strong inhibitory effects on JAK2 kinase activity at micromolar concentrations (not shown). Finally, AZD1480 and ruxolitinib apparently inhibited J53Z1 cells by inducing apoptosis (Figure 8, bottom panel). Additional data demonstrated that inhibited EPO- and SCF-induced activation of ERK1/2, Akt, and STAT5 (not shown). Together, J53Z1 cells provide an excellent cell-based system to screen for JAK2 inhibitors.

## Discussion

In the present study, we generated a JAK2V617F-containing erythroleukemia cell line designated J53Z1. J53Z1 cells belong to the erythroid lineage and are arrested at a stage before proerythroblast during erythroid development. They are positive for surface marker CD71 and negative for TER-119. They express EpoR and c-Kit and are responsive to stimulation by EPO and SCF. They express both GATA1 and GATA2 transcription factors expression thereby representing a unique stage of erythroid development. These cells should have wide applications in basic research to define the regulation of erythroid development and in translational research to identify JAK2 inhibitors for therapeutic drug development.

Identification of JAK2V617F in MPNs has provided an excellent target for drug development. Many potent JAK2 inhibitors have been developed, and some have shown clinical benefits in treatment of myelofibrosis [30], [32], [33]. However, the general outcome of clinical trials of JAK2 inhibitors has been disappointing. Obviously, more effective JAK2 inhibitors are needed. In this regards, J53Z1 cells provide an excellent cell-based system for inhibitor screening as illustrated in Figure 8. In addition, since J53Z1 cells are derived from mice, they can induce leukemia in normal mice. In contrast, existing JAK2V617F-positive human cells (e.g., HEL cells) require immunodeficient mice for implantation. Therefore, J53Z1 cells serve as a better system for testing the efficacy of JAK2 inhibitors *in vivo*.

By establishing a JAK2V617F-positive, p53-null, immortal cell line, our study provides further evidence that JAK2V617F collaborates with loss-of-function of p53 to cause leukemic transformation. Malignant transformation usually involves a gain-of-function mutation of oncogenes and a loss-of-function mutation of tumor suppressor genes. Among various tumor suppressors, p53 is the most frequently mutated [34]-[36]. Interestingly, mutations of p53 are the most common in solid tumors but relatively rare in leukemia [37]. However, many erythroleukemia cell lines identified so far appear to contain p53 mutations. Aside from generating a p53^-/-^ erythroid cell line, our current study also demonstrated the absence of p53 in HCD-57 cells which were derived from a mouse infected at birth with Friend murine leukemia virus [23]. This is consistent with the finding that loss of p53 tumor suppressor function is required for *in vivo* progression of Friend erythroleukemia [38]. Furthermore, our earlier studies identified a M133K p53 mutation in JAK2V617F-positive human erythroleukemia HEL cells [16]. Yet another example of a p53 mutation was found in BCR-Abl-positive human erythroid leukemic K562 cells [39]. These findings support the notion that p53 mutations play a role in erythroblast transformation. Normal p53 suppresses malignant transformation by controlling cell cycle progression, ensuring the fidelity of DNA replication and chromosomal segregation, and inducing apoptosis in response to potentially deleterious events [34]-[37]. It may also have important role in regulating normal proliferation and differentiation of erythroid cells. In this regard, it will be interesting to see if restoration of p53 function in the p53-deficient erythroleukemia can reinstall erythroid differentiation.

## References

[1] Levine RL, Gilliland DG (2008) Myeloproliferative disorders. Blood 112: 2190–2198. 18779404 10.1182/blood-2008-03-077966 PMC2962533

[2] Tefferi A (2008) The history of myeloproliferative disorders: before and after Dameshek. Leukemia 22: 3–13. 17882283 10.1038/sj.leu.2404946

[3] Baxter EJ, Scott LM, Campbell PJ, East C, Fourouclas N, et al (2005) Acquired mutation of the tyrosine kinase JAK2 in human myeloproliferative disorders. Lancet 365: 1054–1061. 15781101 10.1016/S0140-6736(05)71142-9

[4] Levine RL, Wadleigh M, Cools J, Ebert BL, Wernig G, et al (2005) Activating mutation in the tyrosine kinase JAK2 in polycythemia vera, essential thrombocythemia, and myeloid metaplasia with myelofibrosis. Cancer Cell 7: 387–397. 15837627 10.1016/j.ccr.2005.03.023

[5] James C, Ugo V, Le Couedic JP, Staerk J, Delhommeau F, et al (2005) A unique clonal JAK2 mutation leading to constitutive signalling causes polycythaemia vera. Nature 434: 1144–1148. 15793561 10.1038/nature03546

[6] Kralovics R, Passamonti F, Buser AS, Teo SS, Tiedt R, et al (2005) A gain-of-function mutation of JAK2 in myeloproliferative disorders. N Engl J Med 352: 1779–1790. 15858187 10.1056/NEJMoa051113

[7] Zhao R, Xing S, Li Z, Fu X, Li Q, et al (2005) Identification of an acquired JAK2 mutation in polycythemia vera. J Biol Chem 280: 22788–22792. 15863514 10.1074/jbc.C500138200 PMC1201515

[8] Zhao W, Gao R, Lee J, Xing S, Ho WT, et al (2011) Relevance of JAK2V617F positivity to hematological diseases–survey of samples from a clinical genetics laboratory. J Hematol Oncol 4: 4. 21235771 10.1186/1756-8722-4-4 PMC3032761

[9] Xing S, Wanting TH, Zhao W, Ma J, Wang S, et al (2008) Transgenic expression of JAK2V617F causes myeloproliferative disorders in mice. Blood 111: 5109–5117. 18334677 10.1182/blood-2007-05-091579 PMC2384138

[10] Shide K, Shimoda HK, Kumano T, Karube K, Kameda T, et al (2008) Development of ET, primary myelofibrosis and PV in mice expressing JAK2 V617F. Leukemia 22: 87–95. 18033315 10.1038/sj.leu.2405043

[11] Tiedt R, Hao-Shen H, Sobas MA, Looser R, Dirnhofer S, et al (2008) Ratio of mutant JAK2-V617F to wild-type Jak2 determines the MPD phenotypes in transgenic mice. Blood 111: 3931–3940. 18160670 10.1182/blood-2007-08-107748

[12] Mullally A, Lane SW, Ball B, Megerdichian C, Okabe R, et al (2010) Physiological Jak2V617F expression causes a lethal myeloproliferative neoplasm with differential effects on hematopoietic stem and progenitor cells. Cancer Cell 17: 584–596. 20541703 10.1016/j.ccr.2010.05.015 PMC2909585

[13] Marty C, Lacout C, Martin A, Hasan S, Jacquot S, et al (2010) Myeloproliferative neoplasm induced by constitutive expression of JAK2V617F in knock-in mice. Blood 116: 783–787. 20472827 10.1182/blood-2009-12-257063

[14] Akada H, Yan D, Zou H, Fiering S, Hutchison RE, et al (2010) Conditional expression of heterozygous or homozygous Jak2V617F from its endogenous promoter induces a polycythemia vera-like disease. Blood 115: 3589–3597. 20197548 10.1182/blood-2009-04-215848 PMC2867267

[15] Li J, Spensberger D, Ahn JS, Anand S, Beer PA, et al (2010) JAK2 V617F impairs hematopoietic stem cell function in a conditional knock-in mouse model of JAK2 V617F-positive essential thrombocythemia. Blood 116: 1528–1538. 20489053 10.1182/blood-2009-12-259747 PMC3145111

[16] Zhao W, Du Y, Ho WT, Fu X, Zhao ZJ (2012) JAK2V617F and p53 mutations coexist in erythroleukemia and megakaryoblastic leukemic cell lines. Exp Hematol Oncol 1: 15. 23210734 10.1186/2162-3619-1-15 PMC3514099

[17] Shi K, Zhao W, Chen Y, Ho WT, Yang P, et al (2014) Cardiac hypertrophy associated with myeloproliferative neoplasms in JAK2V617F transgenic mice. J Hematol Oncol 7: 25. 24646493 10.1186/1756-8722-7-25 PMC3995113

[18] Jacks T, Remington L, Williams BO, Schmitt EM, Halachmi S, et al (2010) Tumor spectrum analysis in p53-mutant mice. Curr Biol 1994 4: 1–7. 10.1016/s0960-9822(00)00002-6 7922305

[19] Tian C, Gregoli P, Bondurant M (2003) The function of the bcl-x promoter in erythroid progenitor cells. Blood 101: 2235–2242. 12411308 10.1182/blood-2002-04-1217

[20] Zhao AH, Gao R, Zhao ZJ (2011) Development of a highly sensitive method for detection of JAK2V617F. J Hematol Oncol 4: 40. 21985400 10.1186/1756-8722-4-40 PMC3207960

[21] Jin X, Zhao W, Shi K, Ho WT, Zhao ZJ (2013) Generation of a new congenic mouse strain with enhanced chymase expression in mast cells. PLoS One 8: e84340. 24391943 10.1371/journal.pone.0084340 PMC3877308

[22] Roehm NW, Rodgers GH, Hatfield SM, Glasebrook AL (1991) An improved colorimetric assay for cell proliferation and viability utilizing the tetrazolium salt XTT. J Immunol Methods 142: 257–265. 1919029 10.1016/0022-1759(91)90114-u

[23] Ruscetti SK, Janesch NJ, Chakraborti A, Sawyer ST, Hankins WD (1990) Friend spleen focus-forming virus induces factor independence in an erythropoietin-dependent erythroleukemia cell line. J Virol 64: 1057–1062. 2154592 10.1128/jvi.64.3.1057-1062.1990 PMC249217

[24] Chen K, Liu J, Heck S, Chasis JA, An X, et al (2009) Resolving the distinct stages in erythroid differentiation based on dynamic changes in membrane protein expression during erythropoiesis. Proc Natl Acad Sci U S A 106: 17413–17418. 19805084 10.1073/pnas.0909296106 PMC2762680

[25] Kaneko H, Shimizu R, Yamamoto M (2010) GATA factor switching during erythroid differentiation. Curr Opin Hematol 17: 163–168. 20216212 10.1097/MOH.0b013e32833800b8

[26] Sui X, Krantz SB, You M, Zhao Z (1998) Synergistic activation of MAP kinase (ERK1/2) by erythropoietin and stem cell factor is essential for expanded erythropoiesis. Blood 92: 1142–1149. 9694701

[27] Hedvat M, Huszar D, Herrmann A, Gozgit JM, Schroeder A, et al (2009) The JAK2 inhibitor AZD1480 potently blocks Stat3 signaling and oncogenesis in solid tumors. Cancer Cell 16: 487–497. 19962667 10.1016/j.ccr.2009.10.015 PMC2812011

[28] Quentmeier H, Reinhardt J, Zaborski M, Drexler HG (2003) FLT3 mutations in acute myeloid leukemia cell lines. Leukemia 17: 120–124. 12529668 10.1038/sj.leu.2402740

[29] Guo Y, Chen Y, Xu X, Fu X, Zhao ZJ (2012) SU11652 Inhibits tyrosine kinase activity of FLT3 and growth of MV-4-11 cells. J Hematol Oncol 5: 72. 23216927 10.1186/1756-8722-5-72 PMC3524753

[30] Mesa RA, Cortes J (2013) Optimizing management of ruxolitinib in patients with myelofibrosis: the need for individualized dosing. J Hematol Oncol 6: 79. 24283870 10.1186/1756-8722-6-79 PMC4222119

[31] Li Z, Xu M, Xing S, Ho WT, Ishii T, et al (2007) Erlotinib effectively inhibits JAK2V617F activity and polycythemia vera cell growth. J Biol Chem 282: 3428–3432. 17178722 10.1074/jbc.C600277200 PMC2096634

[32] Verstovsek S, Kantarjian H, Mesa RA, Pardanani AD, Cortes-Franco J, et al (2010) Safety and efficacy of INCB018424, a JAK1 and JAK2 inhibitor, in myelofibrosis. N Engl J Med 363: 1117–1127. 20843246 10.1056/NEJMoa1002028 PMC5187954

[33] LaFave LM, Levine RL (2012) JAK2 the future: therapeutic strategies for JAK-dependent malignancies. Trends Pharmacol Sci 33: 574–582. 22995223 10.1016/j.tips.2012.08.005

[34] Ferbeyre G, Lowe SW (2002) The price of tumour suppression? Nature 415: 26–27. 11780097 10.1038/415026a

[35] Petitjean A, Mathe E, Kato S, Ishioka C, Tavtigian SV, et al (2007) Hainaut P, Olivier M. Impact of mutant p53 functional properties on TP53 mutation patterns and tumor phenotype: lessons from recent developments in the IARC TP53 database. Hum Mutat 28: 622–629. 17311302 10.1002/humu.20495

[36] Donehower LA, Lozano G (2009) 20 years studying p53 functions in genetically engineered mice. Nat Rev Cancer 9: 831–841. 19776746 10.1038/nrc2731

[37] Liu Y, Elf SE, Asai T, Miyata Y, Liu Y, et al (2009) The p53 tumor suppressor protein is a critical regulator of hematopoietic stem cell behavior. Cell Cycle 8: 3120–3124. 19755852 10.4161/cc.8.19.9627 PMC4637974

[38] Prasher JM, Elenitoba-Johnson KS, Kelley LL (2001) Loss of p53 tumor suppressor function is required for in vivo progression of Friend erythroleukemia. Oncogene 20: 2946–2955. 11420707 10.1038/sj.onc.1204395

[39] Law JC, Ritke MK, Yalowich JC, Leder GH, Ferrell RE (1993) Mutational inactivation of the p53 gene in the human erythroid leukemic K562 cell line. Leuk Res 17: 1045–1050. 8246608 10.1016/0145-2126(93)90161-d

